# Comparison of Rapid Cytokine Immunoassays for Functional Immune Phenotyping

**DOI:** 10.3389/fimmu.2022.940030

**Published:** 2022-07-04

**Authors:** Anthony S. Bonavia, Abigail Samuelsen, Zissis C. Chroneos, Eric Scott Halstead

**Affiliations:** ^1^ Division of Critical Care Medicine, Department of Anesthesiology and Perioperative Medicine, Penn State Milton S. Hershey Medical Center, Hershey, PA, United States; ^2^ Department of Anesthesiology and Perioperative Medicine, Penn State Milton S. Hershey Medical Center, Hershey, PA, United States; ^3^ Department of Pediatrics, Penn State Milton S. Hershey Medical Center, Hershey, PA, United States; ^4^ Division of Pediatric Critical Care Medicine, Department of Pediatrics, Penn State Milton S. Hershey Medical Center, Hershey, PA, United States

**Keywords:** cytokine, immunoassay, immune phenotype, tumor necrosis factor, interferon gamma

## Abstract

**Background:**

Cell-based functional immune-assays may allow for risk stratification of patients with complex, heterogeneous immune disorders such as sepsis. Given the heterogeneity of patient responses and the uncertain immune pathogenesis of sepsis, these assays must first be defined and calibrated in the healthy population.

**Objective:**

Our objective was to compare the internal consistency and practicality of two immune assays that may provide data on surrogate markers of the innate and adaptive immune response. We hypothesized that a rapid turnaround, microfluidic-based immune assay (ELLA) would be comparable to a dual-color, enzyme-linked immunospot (ELISpot) assay in identifying tumor necrosis factor (TNF) and interferon (IFN)γ production following *ex vivo* whole blood stimulation.

**Design:**

This was a prospective, observational cohort analysis. Whole blood samples from ten healthy, immune-competent volunteers were stimulated for either 4 hours or 18 hours with lipopolysaccharide, anti-CD3/anti-CD28 antibodies, or phorbol 12-myristate 13-acetate with ionomycin to interrogate innate and adaptive immune responses, respectively.

**Measurements and Main Results:**

ELLA analysis produced more precise measurement of TNF and IFNγ concentrations as compared with ELISpot, as well as a four- to five-log_10_ dynamic range for TNF and IFNγ concentrations, as compared with a two-log_10_ dynamic range with ELISpot. Unsupervised clustering accurately predicted the *ex vivo* immune stimulant used for 90% of samples analyzed *via* ELLA, as compared with 72% of samples analyzed *via* ELISpot.

**Conclusions:**

We describe, for the first time, a rapid and precise assay for functional interrogation of the innate and adaptive arms of the immune system in healthy volunteers. The advantages of the ELLA microfluidic platform may represent a step forward in generating a point-of-care test with clinical utility, for identifying deranged immune phenotypes in septic patients.

## Introduction

Sepsis involves a dysregulated, host response to infection that causes life-threatening organ dysfunction (1). Decades of sepsis research and over one hundred failed clinical trials targeting the systemic inflammatory response bear testament to our incomplete understanding of this complex and heterogeneous disease. The current sepsis research paradigm favors the existence of multiple, novel disease endotypes, each with unique immunologic profiles (2, 3). This would offer a plausible explanation for how glucocorticosteroids and other ‘failed’ sepsis therapies may benefit one subset of patients while harming another.

The existence of diverse sepsis endotypes is evidenced by unique transcriptomic signatures, although we currently lack consensus definitions that distinguish between these subsets of patients (2–6). Furthermore, given the time, cost and processing power required to analyze the transcriptome from the blood samples of patients, this approach is not yet ready for clinical, point-of-care implementation. This is a critical shortcoming, as there is a strong correlation between delayed diagnosis/treatment of sepsis and poor patient outcomes (7, 8).

Most transcriptomic investigations relating to sepsis endotypes have concurrently identified high-risk patient populations in whom the innate and/or adaptive immune system is gravely impaired. These same patients also typically have poor disease outcomes (2–6). This observation may offer an opportunity for early risk-stratification of septic patients and their treatment with tailored immune therapies. Assays for the detection of hyper-inflammation, based on high levels of circulating cytokines, are easily performed although they have not yet led to any therapeutic breakthroughs in sepsis. Assays detecting the *absence* of an appropriate immune response in sepsis are more subtle, and thus challenging, to perform. However, they may offer useful information about whether a patient is immunosuppressed and/or likely to respond to immune adjuvant therapy (9, 10). Furthermore, unlike transcriptomic analysis, functional immune assays may be far closer to clinical implementation. The goal of our investigation was to compare the performance of two such assays – the enzyme-linked immunospot (ELISpot) assay and the ELLA microfluidic platform – in measuring surrogate, cytokine markers of the innate and adaptive immune system following *ex vivo* stimulation of whole blood.

The ELISpot uses antibodies to capture and detect analytes of interest that are released by immune cells. Antibody-antigen complexes are visualized as discrete spots, and the one spot-one cell principle allows the sensitive detection of specific subsets of cells while providing information regarding the amount of measured analyte produced per cell (11, 12). ELISpot has been recently shown to be a useful tool for rapid functional immune endotyping, when employed using whole blood from septic patients (9). Key advantages of ELISpot include (i) the ability to quantify cytokines on a per-cell basis, and (ii) the ability to rapidly measure the concentrations of multiple cytokines. These advantages allow the simultaneous interrogation of innate and adaptive arms of the immune system (13, 14). While the ELISpot assay requires minimal technical expertise, its critical shortcoming includes variability in the results it generates (15, 16). Janetzki et al. reported up to 35-fold difference in the processing of identical samples of peripheral blood mononuclear cells by different labs (16). Despite the widespread implementation of Minimal Information about T Cell Assays (MIATA) guidelines to limit inter-lab variability, a recent study demonstrated that at least six replicates with >150 spot forming units (SFU) per well may be needed to provide optimal precision and accuracy for assessing cell mediated immune responses *via* ELISpot (17).

The ELLA immunoassay allows sub-picogram quantification of multiple analytes simultaneously, by employing a pre-calibrated standard curve and automated, microfluidic technology. It does not offer the single-cell resolution of the ELISpot assay and it does not allow the quantification of cytokines on a per cell basis. However, unlike a traditional ELISA or the ELISpot, it is rapid and requires minimal user intervention, thus reducing bias and the opportunity for user error. We hypothesized that, as compared with ELISpot, the ELLA assay would offer single-step, rapid and highly reproducible information about a patient’s functional immune response following *ex vivo* stimulation of whole blood samples.

## Materials, Equipment and Methods

### Study Design

This observational study was performed on healthy, adult volunteers. All persons were enrolled at the Penn State Milton S Hershey Medical Center (Hershey, PA), following study approval by the Human Study Protection Office (Institutional Review Board Approval #15328 and 10357) and after obtaining informed consent. To minimize the potential for confounding effects, we excluded volunteers having active hematologic malignancies (leukemia or lymphoma), diagnosed autoimmune disorders and those who were on immunomodulating therapies.

### Processing of Blood Samples

Two milliliters of venous blood were collected in tubes containing sodium heparin. They were kept at room temperature until time of processing. Leukocyte count and cellular differential was determined from whole blood collected in a tube containing ethylenediamine tetraacetic acid (EDTA).

### Preparation of ELISpot Assay for the Assessment of Innate and Adaptive Immune Function

ELISpot analysis was used to assess innate immune function by measuring the production of tumor necrosis factor (TNF) following *ex vivo* stimulation of whole blood, and to assess adaptive immune function by measuring the production of interferon (IFN) γ (9). Both assays were performed following 4 hour and 18 hour incubation at 37°C and 5% CO_2_, based on a recent report of ELISpot for functional immune phenotyping in sepsis that utilized an 18 h incubation time (9). Double-color, enzymatic-based ELISpot plates allowed the simultaneous measurement of individual cytokines in each blood sample, as well as identifying those cells which produce both TNF and IFNγ. We chose dual-color ELISpot to assess the feasibility of single-step quantification of TNF and IFNγ and compare it with the single-step ELLA assay.

Polyvinylidene difluoride strip plates were activated with ethanol, rinsed and then incubated overnight with capture antibodies, per manufacturer’s instructions (ImmunoSpot, Cellular Technology, Cleveland, OH). 50 *µ*l of whole blood was diluted ten-fold in media containing one of three stimulants: (1) 500 ng/ml of anti-CD3 (Cat# 300302, Biolegend, San Diego, CA) with 2.5 *µ*g/ml anti-CD28 (Cat# 302902, Biolegend), (2) 80 nM (49.3 ng/ml) phorbol 12-myristate 13-acetate (PMA) with 1.3 *µ*M (0.97 *µ*g/ml) ionomycin (Thermo Fisher Scientific, Waltham, MA), or (3) 500 ng/ml lipopolysaccharide (LPS) from *Salmonella enterica* strain abortus equi (Cat# L1887, Sigma-Aldrich, St. Louis, MO). Concentrations of anti-CD3/anti-CD28 and LPS were determined based on previous reports of their use in similar experimental contexts (9, 18, 19). PMA/ionomycin concentration was determined based on results of preliminary assays, and on comparable results obtained by using premixed Cell Stimulation Cocktail (Cat# 00-4970-03, Thermo Fisher) (**Supplementary Figure S1**).

Samples were run in duplicate for each test condition. Following the incubation period, anti-human IFNγ and anti-human TNF detection antibodies, conjugated to fluorescein isothiocyanate (FITC) and biotin, respectively, were added to each well and incubated for 2 hours. Tertiary solutions of anti-FITC horseradish peroxidase and streptavidin alkaline phosphatase were subsequently added, followed by blue developer solution for TNF spots and red developer solution for IFNγ spots, per manufacturer’s instructions.

### ELISpot Analysis

Samples were scanned for spot count and intensity using a Cellular Technology series 6 Immunospot Universal Analyzer with ImmunoSpot 7.0 program (Cellular Technology Analyzers, Shaker Heights, OH). Optimal spot detection parameters were determined following aggregate review of all ELISpot images. Once detection parameters were optimized, spot counts were determined in a fully automated and blinded fashion. Quality control was performed, after all images were acquired, to remove any artifacts that could be inadvertently counted by the analyzer as spots.

### Evaluation and Standardization of Cytokine Production by ELISpot Analysis

Cytokine production was reported either as SFU per 50 *µ*l of whole blood stimulated *ex vivo*. Each SFU represented one cytokine-secreting cell (producing IFNγ only, TNF only, or both IFNγ and TNF). In addition to the number of cytokine-producing cells, data are reported using an automated analytic method (Cellular Technology ImmunoSpot 7.0 Software) based upon the pixel density/intensity of each ELISpot well. Mean spot size (MSS) was reported, and mean spot intensity (MSI) and total well intensity (TWI) were calculated by the software based on measured parameters (9, 13, 20). MSI is the arithmetic mean of the intensity function values of the counted image for the spot pixels, multiplied by 1000 for better precision. TWI is the MSI multiplied by MSS, and as such represent the total amount of “color” per spot.

### ELLA Microfluidic Immunoassay

Whole blood (50 *µ*L) was added to 450 μL of HEPES-buffered RPMI media, in a 1.6 ml polypropylene tube containing one of three stimulants: (1) 500 ng/ml anti-CD3 with 2.5 *µ*g/ml anti-CD28, (2) 16 nM phorbol 12-myristate 13-acetate (PMA) with 1.3 *µ*M ionomycin, or (3) 500 pg/ml LPS from *Salmonella enterica* strain abortus equi. Blood was incubated at 37°C for either 4 hours or 18 hours, resulting in a total of six samples per patient. Following incubation, samples were centrifuged at 1000 x g for 5 minutes and the supernatant was frozen at -80°C until processing.

Supernatants from *ex vivo* blood stimulation were analyzed for the presence of three cytokines: IFNγ, IL-6 and TNF, by using Simple Plex assays run on the ELLA microfluidic immunoassay system (ProteinSimple, San Jose, CA). Supernatants were diluted at a 1:1 ratio with sample diluent, and 50 μl of this solution was added to each sample inlet on the ELLA cartridge, per manufacturer’s instruction. Wash buffer was added to the appropriate wells on the ELLA cartridge. Sample results were reported using Simple Plex Runner v.3.7.2.0 (ProteinSimple) and were available approximately 90 minutes after initiation of the run start.

### Unsupervised Sample Clustering

To validate the ability of each analysis to discern stimulation conditions unsupervised clustering analysis of ELLA and ELISpot results was performed. Cytokine concentrations derived from Simple Plex Runner and Immunospot were analyzed using JMP Pro 16.0.0 (SAS Institute Inc., Cary, NC). Ward’s method of hierarchical clustering was utilized to ascertain whether the program could identify the type of stimulus and the time of incubation based on the plasma concentrations of IFNγ, IL-6 and TNF (ELLA) and SFU, TWI and MSI for IFNγ and TNF (ELISpot). K means clustering was also performed on these data sets, using a predefined cluster size of 6, which is the number of different stimulation conditions used (anti-CD3/anti-CD28, PMA/ionomycin and LPS, each for 4 hours and 18 hours).

## Results

### Human Cohort Data

Ten healthy volunteers had a mean age of 39 (range 25 to 53 years old), with 4 subjects identifying as female. Eight subjects were Caucasian, one was Hispanic, and one was of Asian race. Mean leukocyte count was 5.9 (± 0.8) x 10^3^/*µ*l, mean lymphocyte count was 1.9 (± 3.9) x 10^3^/ *µ*l and mean monocyte count was 0.5 (± 0.1) x 10^3^/ *µ*l of blood.

### ELISpot Intra-Assay Variability and Cytokine Response to Immune Stimulants

**Figure 1:**
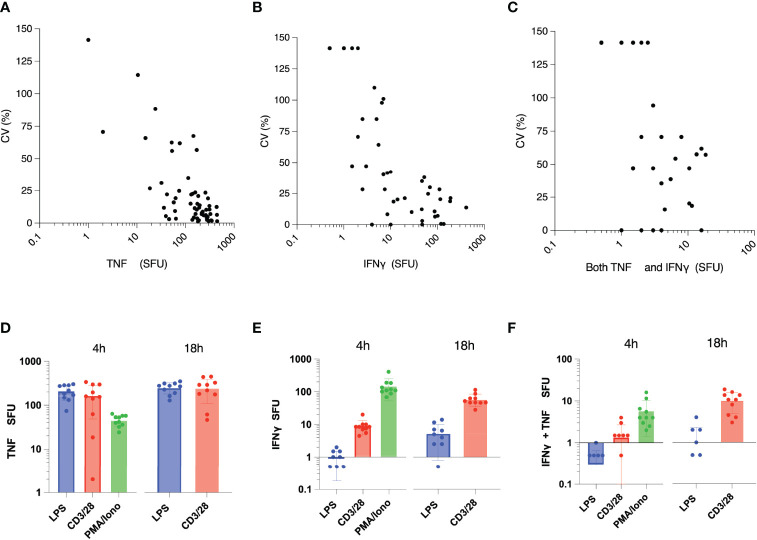
Intra-assay variability of ELISpot immunoassay, assessed by percentage coefficient of variation (CV) for: **(A)** Tumor Necrosis Factor (TNF), **(B)** Interferon (IFN)γ, and **(C)** dual-staining cells (both IFNγ and TNF). **(D)** TNF, **(E)** IFNγ, and **(F)** dual-cytokine production following *ex vivo* stimulation with various immune stimulants (x-axis) for 4 hours or 18 hours. N = 2 for each measured analyte. 18 hour ELISpot data includes response to LPS and anti-CD3/anti-CD28 only, due to cell over-stimulation with PMA. SFU, spot forming units.

ELISpot samples were run in duplicate, with mean SFU coefficient of variation (CV%) of 57% for IFNγ (IQR 11% - 107%), 21% for TNF (IQR 4.7% - 23%) and 50% for dual color spots (IQR 0 – 94%) (**Figures 1A–C**). Mean MSI coefficient of variation was 4.8% for IFNγ (IQR 2% - 8%) and 4% for TNF (IQR 2% - 6%). Mean TWI coefficient of variation was 19% for IFNγ (IQR 6% - 28%) and 24% for TNF (IQR 9% - 33%). At the doses of stimulants used, the highest measured value for SFU was 477 for TNF and 448 for IFNγ. The ELISpot reproducibility was lower with decreasing cytokine concentrations, with CV rapidly increasing to >50% at less than 20 SFU.

As expected, LPS stimulation primarily increased TNF production. There was minimal incremental cytokine production between 4 hours and 18 hours, indicating saturation of TNF production by 4 hours post-stimulation (**Figure 1D**). Large amounts of TNF were also produced by anti-CD3/CD28 stimulation, as previously described (21), although production of this cytokine was also saturated by 4 hours of stimulation.

IFNγ was produced in significant quantities by both anti-CD3/CD28 and PMA/ionomycin stimulation (**Figure 1E**). At the doses of anti-CD3/28 used, IFNγ was still actively being produced at 18 hours following stimulation. Conversely, PMA/ionomycin production produced robust (but countable) cytokine spot production after 4 hours, while 18 hours of stimulation produced red spots that were too numerous to count (confluent, red colored wells). Given the dual-color ELISpot assay utilized in these experiments, this result also made it impossible to accurately quantify the number of TNF producing cells with this stimulant at this time point (**Figure 1F**).

### ELLA Intra-Assay Variability and Cytokine Response to Immune Stimulants

**Figure 2:**
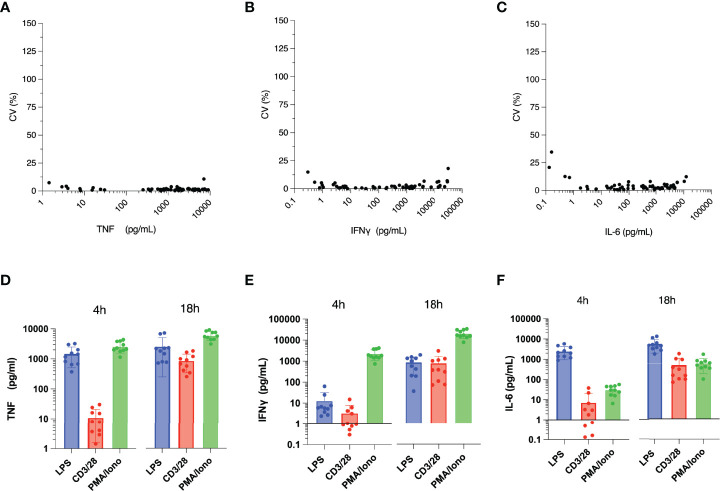
Intra-assay variability of ELLA microfluidic immunoassay, assessed by percentage coefficient of variation (CV) for: **(A)** Tumor Necrosis Factor (TNF), **(B)** Interferon (IFN)γ, and **(C)** Interleukin (IL)-6. **(D)** TNF, **(E)** IFNγ, and **(F)** IL-6 production following *ex vivo* stimulation with various immune stimulants (x-axis) for 4 hours or 18 hours. N = 3 for each measured analyte.

The ELLA instrument analyzes up to four analytes simultaneously and measures each sample in triplicate. The intra-assay variabilities for each analyte are shown in **Figures 2A–C**. Mean coefficient of variation was for 2.8% IFNγ [Interquartile range (IQR) 0.9% - 2.4%], 1.8% for TNF (IQR 0.9% - 2.0%) and 4.2% for IL-6 (IQR 1.7% - 4.5%). As expected, CVs were higher at the low (<1 pg/mL) and high (>10,000 pg/mL) limits of cytokine concentrations. The CVs for ELLA within the manufacturer’s recommended range were very low. The lower limits of quantification were 0.17, 0.28 and 0.3 pg/ml for IFNγ, IL-6 and TNF respectively. The upper limits of quantification were 4000, 6630 and 1160 pg/ml for IFNγ, IL-6 and TNF respectively.

**Table 1** reports the concentrations of cytokines produced following *ex vivo* stimulation of whole blood under different conditions. As expected from known innate immune response mechanisms, stimulation with LPS produced concentrations of TNF and IL-6 that were highly correlated following 4 hours (R^2^ = 0.92, P<0.0001) and 18 hours (R^2^ = 0.95, P<0.0001) of stimulation (**Figures 2D–F**). TNF concentrations following 4 hours of stimulation with LPS were highly correlated with those at 18 hours of stimulation (R^2^ = 0.81, P<0.0004) (**Figure 2D**). The congruence of this pattern of cytokine production with those obtained by ELISpot (**Figure 1D**) indicates that the type and dose of LPS used for our experiments may be compatible with point-of-care testing. The same was not true of IFNγ production following 4 hours versus 18 hours of either anti-CD3/anti-CD28 or PMA/ionomycin stimulation (**Figure 2E**). This may have been, in part, due to the low plasma IFNγ concentration following 4 hours of anti-CD3/anti-CD28 stimulation (mean 3 pg/ml, IQR 1 – 4 pg/ml) and the high IFNγ concentration following 18 hours of PMA stimulation (mean 19688 pg/ml, IQR 14346 – 27935 pg/ml).

**Table 1:** Plasma cytokine concentrations, measured by ELLA microfluidic assay, following stimulation of whole blood in 10 healthy patients.

Immune Stimulant	Duration of stimulation (hours)	Cytokine measured	Mean cytokine Concentration (pg/ml)	Interquartile range (pg/ml)
Anti-CD3/anti-CD28	4	IFNγ	3	1 – 4
		TNF	11	3 – 16
		IL-6	8	1 – 6
Anti-CD3/anti-CD28	18	IFNγ	742	153 - 1041
		TNF	871	447 – 1129
		IL-6	472	108 – 746
PMA/ionomycin	4	IFNγ	2257	1506 – 3310
		TNF	2634	1943 – 3598
		IL-6	32	19 – 45
PMA/ionomycin	18	IFNγ	19688	14346 – 27935
		TNF	5978	4535 – 7421
		IL-6	590	349 – 700
LPS	4	IFNγ	13	4 – 7
		TNF	1492	7133 – 1902
		IL-6	2588	1388 – 3513
LPS	18	IFNγ	824	246 – 1280
		TNF	2594	953 – 2263
		IL-6	5270	3145 – 5545

### Correlation Between ELLA and ELISpot Measurements

The ELISpot assay yields multiple measurements; while SFU denotes the number of cytokine-producing cells, MSI and TWI provide additional information regarding the pattern of cytokine production. Equivalent parameters cannot be derived from enzyme-linked immunosorbent assay (ELISA) or ELLA analysis. We hypothesized these additional ELISpot parameters may be related to the SFU, that is, that stimulation resulting in more SFU would also result in more intense cytokine production per cell.

**Figure 3:**
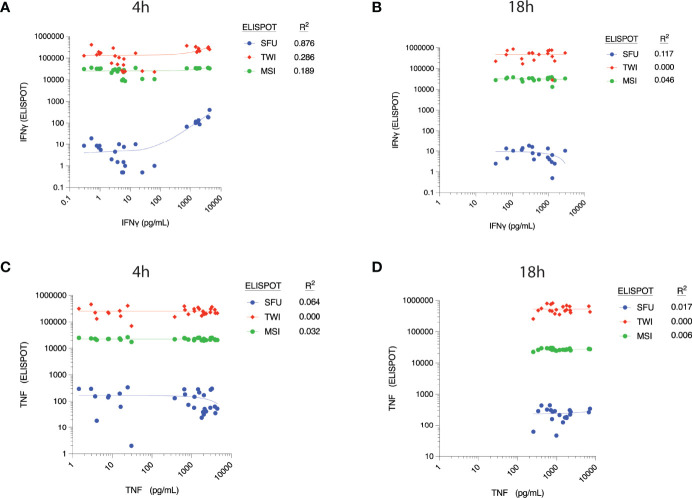
Correlation between cytokine concentrations, as measured by ELLA microfluidic immunoassay (x-axis), and different parameters measured by ELISpot assay at 4h post-*ex vivo* stimulation. Measured plasma cytokine concentration of Interferon (IFN) γ versus corresponding ELISpot spot forming units (SFU), Total Well Intensity (TWI) and Mean Spot Intensity (MSI) at **(A)** 4 hours and **(B)** 18 hours of stimulation. Measured plasma cytokine concentration of Tumor Necrosis Factor (TNF) versus corresponding ELISpot spot forming units (SFU), Total Well Intensity (TWI) and Mean Spot Intensity (MSI) at **(C)** 4 hours and **(D)** 18 hours of stimulation.

There appeared to be a correlation between ELLA-measured IFNγ concentration and SFU at 4h (R^2^ = 0.876, **Figure 3A**), although concentrations did not correlate with TWI and MSI at this time point. The correlation between IFNγ concentration and ELISpot parameters at 18h was weak, as was the correlation between TNF concentrations and ELISpot parameters at both time points (**Figures 3B–D**).

We further investigated whether there was a direct relationship between absolute monocyte/lymphocyte count and IFNγ/TNF production, as measured by ELLA versus ELISpot assays. Absolute monocyte count was moderately correlated with TNF production following LPS stimulation, as measured by ELLA analysis (R^2^ = 0.68) (**Supplementary Table 1**, and **Supplementary Figure S2**). It correlated poorly with TNF SFU by ELISpot analysis (R^2^ = 0.34). Absolute lymphocyte count was not significantly correlated with IFNγ or TNF concentrations.

### Unsupervised Sample Clustering

Establishing a predictable physiologic response when whole blood is stimulated is important to be able to identify normal versus deranged immunologic function. To validate the ability of ELLA and ELISpot assays to predict a ‘normal’ functional immune response, we performed hierarchical and non-hierarchical clustering of samples by aggregate cytokine concentrations. The goal was to investigate whether *ex vivo* stimulation with each of the three stimulants (LPS, PMA/ionomycin or anti-CD3/anti-CD28 antibodies) would produce cytokine signatures that were unique enough to be identified by machine learning. A reproducible cytokine response in healthy patients may allow this same approach to be utilized in fully automated, immune phenotyping of patients having a dysregulated immune response, such as septic patients.

**Figure 4:**
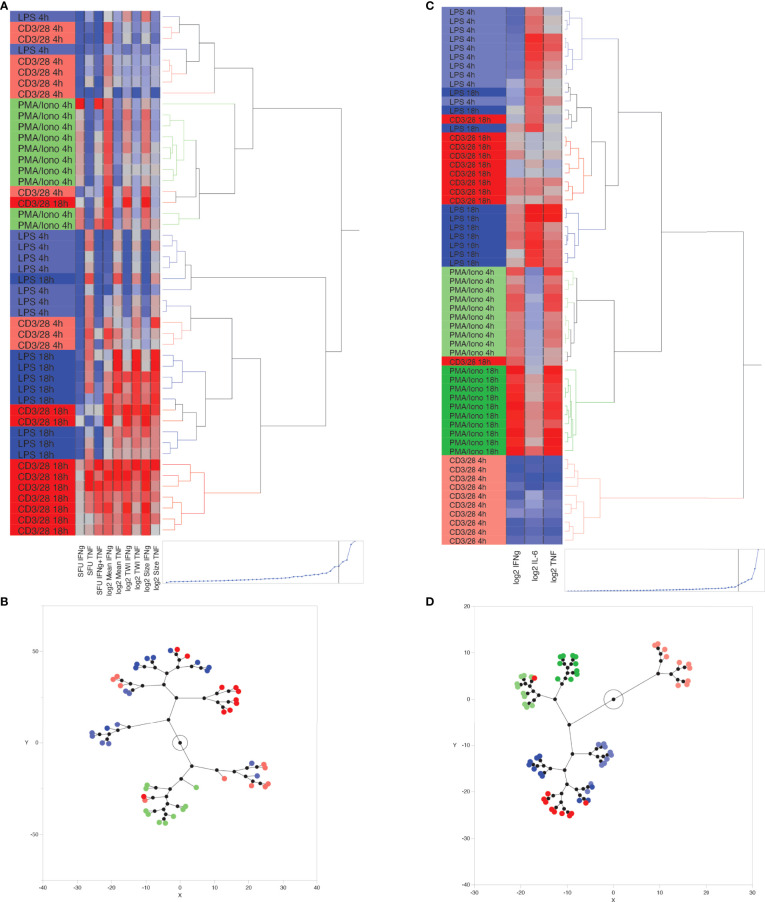
Unsupervised, hierarchical cluster analysis using Ward’s minimum variance method, to predict *ex vivo* stimulant by using ELLA versus ELISpot data from 10 healthy volunteers. **(A)** Dendrogram with heat map demonstrating hierarchical clustering by using ELISpot-derived SFU, log_2_-TWI, MSI and MSS, **(B)** Constellation plot showing data derived from **Figure 4B**, **(C)** Dendrogram with heat map demonstrating hierarchical clustering by using ELLA-derived, log_2_-transformed, plasma cytokine concentrations for Interferon (IFN)γ, Tumor Necrosis Factor (TNF), and interleukin (IL)-6, **(D)** Constellation plot showing data derived from **Figure 4C**.

Unsupervised hierarchical clustering of ELISpot-derived IFNγ, TNF and dual (IFNγ + TNF) SFU appropriately allocated 54% of stimulant conditions. A sample was considered appropriately allocated to a cluster when JMP software grouped it together with the largest contiguous cluster of identically stimulated samples (stimulant type and duration of stimulation were the two cluster-defining variables). Since PMA/ionomycin-stimulated wells produced confluent spots, which precluded reliable measurement of IFNγ or TNF SFU, this data was excluded from the clustering process. K means clustering, based on SFU, confirmed these results by appropriately allocating 56% of samples when 5 clusters (corresponding to the 5 known stimulation conditions) were predefined. Next, we investigated whether the addition of log_2_-transformed TWI, MSI and/or MSS data to the number of SFU improved inter-cluster discrimination. We found that appropriate clustering improved to 72% when including all dual-color ELISpot parameters (**Figures 4A, B**), although appropriate K-means clustering, using this same data, remained at 56%. The combined use of SFU with log_2_-transformed TWI, MSI and MSS (**Figures 4A, B**) was thus considered the optimal method of clustering by ELISpot, since removal of any one of these parameters worsened the number of appropriately clustered samples.

Hierarchical clustering based on log_2_-transformed IL-6, IFNγ, TNF concentrations, as measured by the ELLA platform, appropriately clustered 90% of samples using 5 or fewer hierarchical decision nodes (**Figures 4C, D**). K means clustering based on IL-6, IFNγ, TNF concentrations, and by predefining 6 clusters, appropriately identified 73% of stimulated samples, with most clustering errors occurring based on the proximity of cluster distances assigned to anti-CD3/anti-CD28-stimulated samples at 4 hours and 18 hours of stimulation.

## Discussion

The timely diagnosis and management of sepsis has historically been confounded by changing disease definitions and highly heterogeneous disease presentation. Multiple studies over the past decade have demonstrated between two to four subtypes of sepsis, each associated with a characteristic innate and/or adaptive immune response (2–6). As these responses have profound prognostic implications, the next step in improving the clinical care of septic patients hinges on the timely identification of these subtypes. In this study, we report proof of principle results on the use of the ELLA microfluidic platform to rapidly and accurately quantify innate and adaptive immune function, and we compare it to the established ELISpot platform in healthy volunteers (9). While this type of approach has typically been used in the study of sepsis, it may also be useful in other acute inflammatory disorders such as rheumatologic conditions or transplantation, just to mention a few. We show that the ELLA-based immunoassay has a lower coefficient of variation for all measured cytokines, one which is an order of magnitude lower than equivalent parameters measured on ELISpot analysis (i.e., SFU, TWI and MSI).

The ELLA offers several additional advantages that make it attractive as a point of care test. It has a faster turnaround time, taking only 90 minutes for sample analysis, as compared with approximately 3 hours for dual-color ELISpot analysis. The ELLA platform is also fully automated, requiring only the addition of sample supernatant to the cartridge prior to measurement. By contrast, the ELISpot assay requires two steps which may cause significant result variability. The first includes calibration of the ELISpot assay, that is, user-based definition of what is to be counted as a spot by the imaging software. The second is the requirement for a quality control step to remove image artifacts that may be inadvertently counted as spots.

The central goal of the current study was to develop a reproducible assay that appropriately identifies a physiologic (‘normal’) immune response to *ex vivo* stimulation. LPS binds to toll-like receptor 4 and triggers an intracellular MyD88-dependent signaling cascade that results in the transcription of proinflammatory genes and the production of IL-1 and TNF by innate immune cells such as monocytes (22–25). CD3 and CD28 receptor co-stimulation activate T cells *via* the T cell receptor complex. This mimics the binding of T cells (surrogates of adaptive immune system) to antigen presenting cells, a process that activates resting T cells and triggers the production of IFNγ We chose an additional stimulant of the adaptive immune system, based on preliminary data demonstrating a robust cytokine response within 4 hours. This stimulant was PMA combined with ionomycin. PMA activates T cells by bypassing the T cell receptor complex signaling mechanism. In the presence of the calcium-binding ionophore, ionomycin, PMA activates AKT causing T cell activation including cell proliferation and cytokine production.

While the concentrations of PMA and ionomycin selected for this study caused robust IFNγ production at 4 hours, they also caused overstimulation (and possibly activation-induced T cell death) of whole blood when continuously exposed for 18 hours. This occurred in all ten healthy volunteers, rendering it impossible to discern discrete TNF- or IFNγ-producing cells at 18 hours following PMA stimulation by ELISpot analysis. Thus, we hypothesize that an inability to mount a T cell response following PMA stimulation for 4 hours (and especially for 18 hours) might indicate profound T cell immunoparalysis. This theory needs to be tested in future investigations involving septic or immune-compromised patients. We were unable to discern a fixed concentration of PMA and ionomycin that caused appropriate (but not over-) stimulation of cells both at 4 hours and 18 hours. Based on our goal to move toward a short turnaround-time, point of care test, we thus tailored our stimulant doses to optimize the number of countable SFU at 4 hours.

Like ELISA assays, the ELLA platform provides aggregate information about cytokine production without providing any indication of the heterogeneity of cytokine production in the cell population. ELISpot provides more granular information about the amount of cytokine production per cell. However, as it relies on image-based quantitation, the tradeoff of this assay includes (1) image artifacts which, if significant enough, may completely obscure the collection of any meaningful data and (2) a limited dynamic range, since robust cell stimulation in a limited well area will lead to the inability to distinguish discrete SFUs. This limitation appears to be especially pronounced when using a dual-color ELISpot assay as every stained portion of the assay membrane may only stain red, blue or purple but not all at once. We discovered the limit of accurate spot discrimination was approximately 500 red or blue SFU. Numbers in excess of these parameters created problems in distinguishing individual cytokine-secreting cells. This limitation may be alleviated by utilizing single-color ELISpot assays and separately quantifying TNF or IFNγ-producing cells. However, this increases the cost and processing time of the assay while still producing values that, at best, are three log_10_ scales smaller than the ELLA assay and thus more prone to inter-assay variability. Conversely, the ELLA’s larger dynamic range is also the most likely reason why it outperforms the ELISpot assay when results are used for unsupervised clustering analysis.

In conclusion, by using the ELLA platform, we have introduced a rapid and reproducible, whole blood functional assay for simultaneously interrogating the innate and adaptive immune response. Furthermore, we have defined the ranges of normal cytokine secretion by leukocytes from healthy volunteers following *ex vivo* stimulation of whole blood. These ranges of normal cytokine secretion will allow the assay to be leveraged in future clinical investigations of patients with suspected immune dysfunction, such as sepsis.

## Data Availability Statement

The raw data supporting the conclusions of this article will be made available by the authors, without undue reservation.

## Ethics Statement

The studies involving human participants were reviewed and approved by Penn State Human Subjects Protection Office. The patients/participants provided their written informed consent to participate in this study.

## Author Contributions

AB: Conceptualization, Methodology, Formal Analysis, Funding Acquisition, Investigation, Writing Original Draft, Review and Editing, Project Administration. AS: Investigation, Data Curation, Writing - Review and Editing. ZC: Resources, Writing - Review and Editing. EH: Conceptualization, Methodology, Formal Analysis, Resources, Writing - Original Draft, Review and Editing. All authors contributed to the article and approved the submitted version.

## Funding

This work was funded by the National Institute of General Medical Sciences grant# K08GM138825 (AB).

## Conflict of Interest

The authors declare that the research was conducted in the absence of any commercial or financial relationships that could be construed as a potential conflict of interest.

## Publisher’s Note

All claims expressed in this article are solely those of the authors and do not necessarily represent those of their affiliated organizations, or those of the publisher, the editors and the reviewers. Any product that may be evaluated in this article, or claim that may be made by its manufacturer, is not guaranteed or endorsed by the publisher.

## Supplementary Material

Supplementary Figure 1 Dose-response relationship for interferon γ production (as measured by SFU on ELISpot), following PMA and ionomycin stimulation for **(A)** 4 hours and **(B)** 18 hours of stimulation.

Supplementary Figure 2 Correlation between cell count and cytokine concentrations following *ex vivo* stimulation, as measured by ELLA and ELISpot analysis. Absolute lymphocyte count versus IFNγ concentration (ELLA) and IFNγ SFU (ELISpot) following **(A)** 4 hours and **(B)** 8 hours of stimulation. Absolute lymphocyte count versus TNF concentration (ELLA) and TNF SFU (ELISpot) following **(C)** 4 hours and **(D)** 8 hours of stimulation. Absolute monocyte count versus TNF concentration (ELLA) and TNF SFU (ELISpot) following **(E)** 4 hours and **(F)** 8 hours of stimulation.
