# Supplementary figures and images for: Comparison of Rapid Cytokine Immunoassays for Functional Immune Phenotyping
Source: Front Immunol. 2022 Jul 4;13:940030. doi: 10.3389/fimmu.2022.940030 (PMC9289684; doi:10.3389/fimmu.2022.940030)

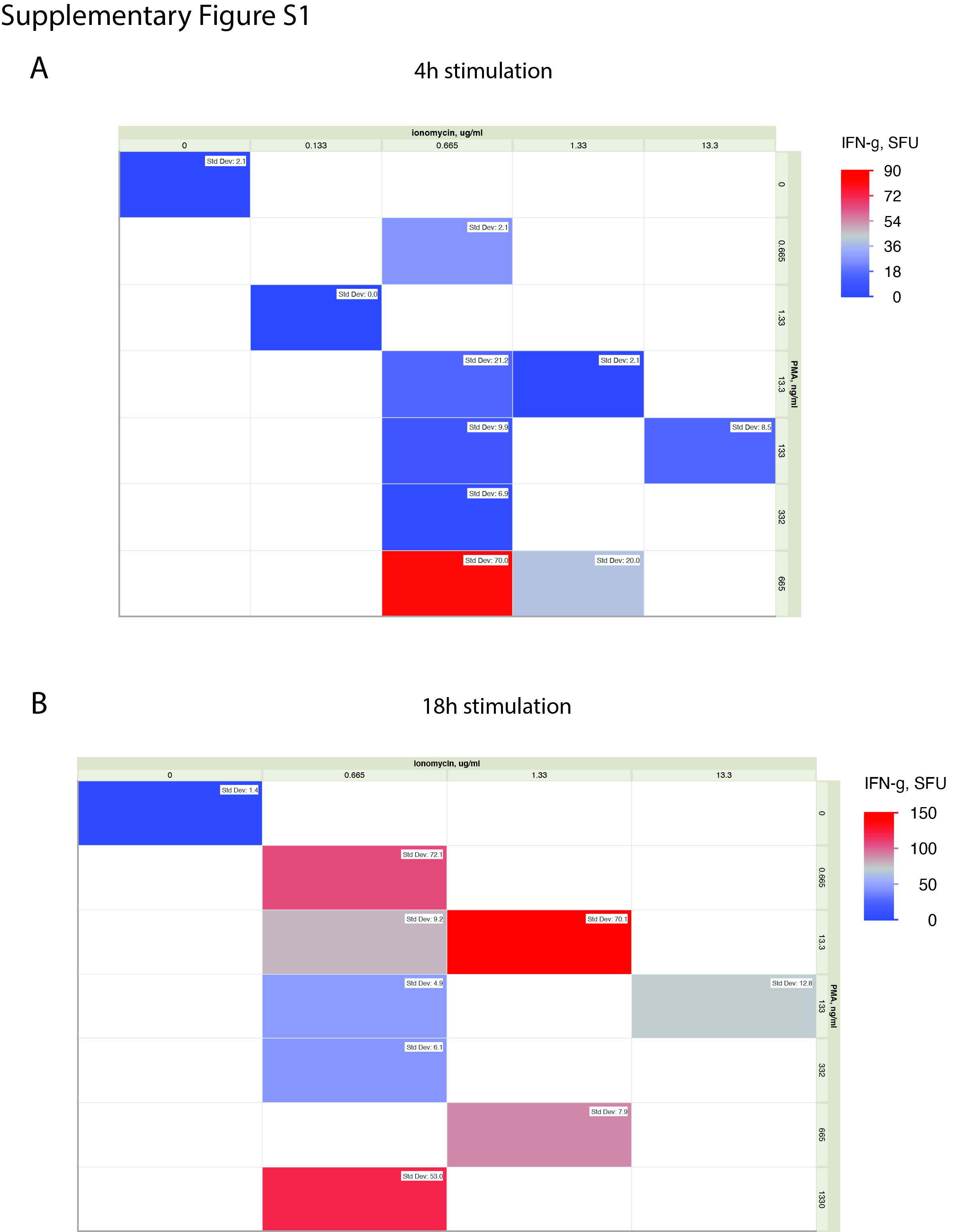

Supplement: Supplementary Figure 1 — Dose-response relationship for interferon γ production (as measured by SFU on ELISpot), following PMA and ionomycin stimulation for (A) 4 hours and (B) 18 hours of stimulation. [file Image_1.jpeg]

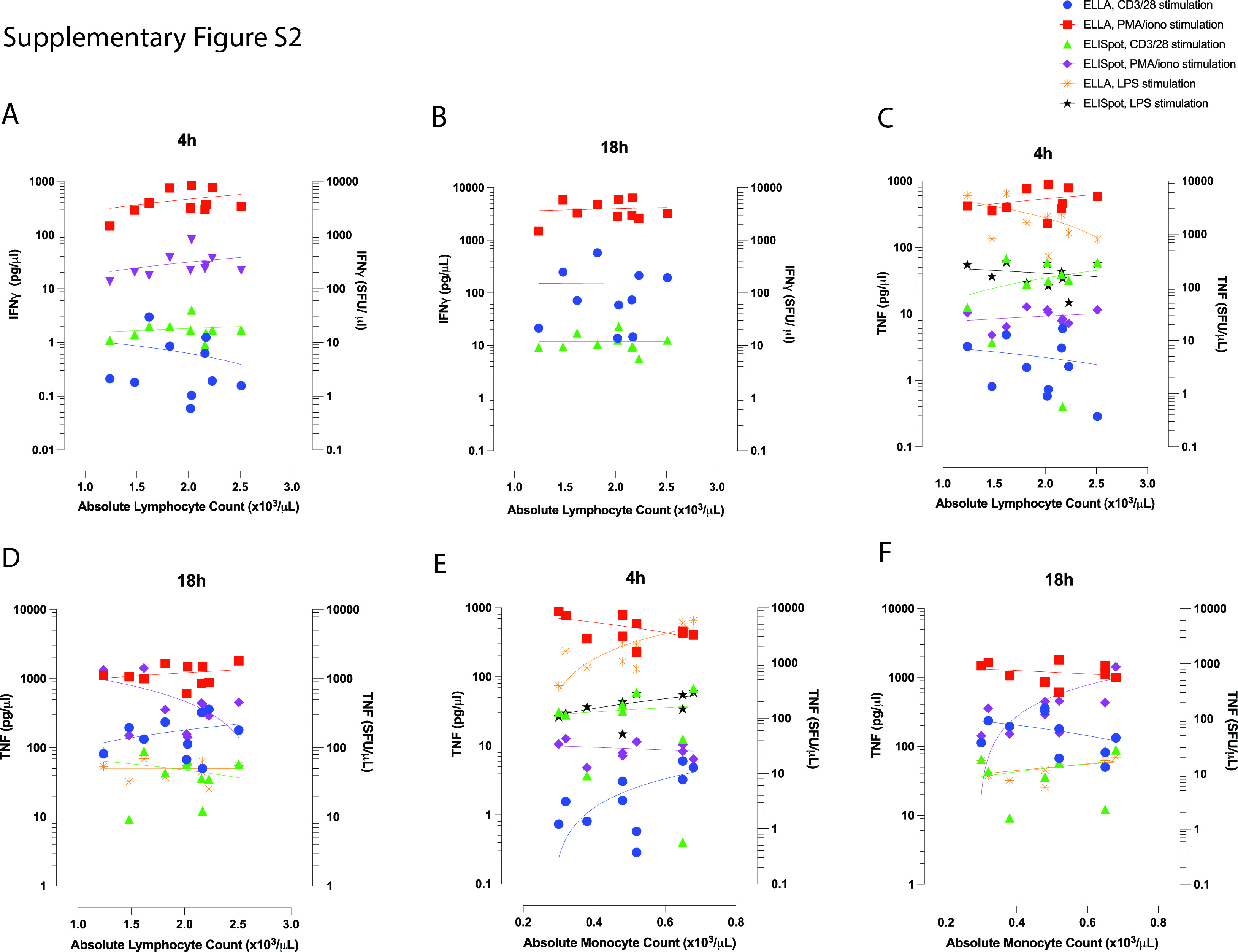

Supplement: Supplementary Figure 2 — Correlation between cell count and cytokine concentrations following ex vivo stimulation, as measured by ELLA and ELISpot analysis. Absolute lymphocyte count versus IFNγ concentration (ELLA) and IFNγ SFU (ELISpot) following (A) 4 hours and (B) 8 hours of stimulation. Absolute lymphocyte count versus TNF concentration (ELLA) and TNF SFU (ELISpot) following (C) 4 hours and (D) 8 hours of stimulation. Absolute monocyte count versus TNF concentration (ELLA) and TNF SFU (ELISpot) following (E) 4 hours and (F) 8 hours of stimulation. [file Image_2.jpeg]
