# Supplementary material for: Comparison of Rapid Cytokine Immunoassays for Functional Immune Phenotyping
Source: Front Immunol. 2022 Jul 4;13:940030. doi: 10.3389/fimmu.2022.940030 (PMC9289684; doi:10.3389/fimmu.2022.940030)
Supplement: Supplementary file 3 [file Table_1.docx]

Supplementary Material

**Supplementary Table 1.** Relationship between cell count and cytokine production, as measured by R-squared for ELLA and ELISpot assays.

| **Assay** | **Cell count** | **Correlation with cytokine** | **Stimulant** | **Duration of Stimulation (h)** | **R^2^** |
| --- | --- | --- | --- | --- | --- |
| ELLA | Absolute lymphocyte count | IFNγ | CD3/28 | 4 | 0.04 |
| ELLA | Absolute lymphocyte count | IFNγ | PMA/ionomycin | 4 | 0.10 |
| ELISpot | Absolute lymphocyte count | IFNγ | CD3/28 | 4 | 0.02 |
| ELISpot | Absolute lymphocyte count | IFNγ | PMA/ionomycin | 4 | 0.07 |
| ELLA | Absolute lymphocyte count | IFNγ | CD3/28 | 18 | 7.4 X 10^-5^ |
| ELLA | Absolute lymphocyte count | IFNγ | PMA/ionomycin | 18 | 0.01 |
| ELISpot | Absolute lymphocyte count | IFNγ | CD3/28 | 18 | 5.6 X 10^-6^ |
| ELLA | Absolute lymphocyte count | TNF | CD3/28 | 4 | 0.04 |
| ELLA | Absolute lymphocyte count | TNF | PMA/ionomycin | 4 | 0.1 |
| ELISpot | Absolute lymphocyte count | TNF | CD3/28 | 4 | 0.1 |
| ELISpot | Absolute lymphocyte count | TNF | PMA/ionomycin | 4 | 0.06 |
| ELLA | Absolute lymphocyte count | TNF | LPS | 4 | 0.3 |
| ELISpot | Absolute lymphocyte count | TNF | LPS | 4 | 0.05 |
| ELLA | Absolute lymphocyte count | TNF | CD3/28 | 18 | 0.09 |
| ELLA | Absolute lymphocyte count | TNF | PMA/ionomycin | 18 | 0.06 |
| ELISpot | Absolute lymphocyte count | TNF | CD3/28 | 18 | 0.09 |
| ELLA | Absolute lymphocyte count | TNF | LPS | 18 | 0.29 |
| ELISpot | Absolute lymphocyte count | TNF | LPS | 18 | 2.5 X 10^-5^ |
| ELLA | Absolute monocyte count | TNF | CD3/28 | 4 | 0.51 |
| ELLA | Absolute monocyte count | TNF | PMA/ionomycin | 4 | 0.31 |
| ELISpot | Absolute monocyte count | TNF | CD3/28 stimulation | 4 | 0.02 |
| ELISpot | Absolute monocyte count | TNF | PMA/ionomycin | 4 | 0.05 |
| ELLA | Absolute monocyte count | TNF | LPS | 4 | 0.68 |
| ELISpot | Absolute monocyte count | TNF | LPS | 4 | 0.34 |
| ELLA | Absolute monocyte count | TNF | CD3/28 | 18 | 0.15 |
| ELLA | Absolute monocyte count | TNF | PMA/ionomycin | 18 | 0.06 |
| ELISpot | Absolute monocyte count | TNF | CD3/28 | 18 | 0.11 |
| ELLA | Absolute monocyte count | TNF | LPS | 18 | 0.53 |
| ELISpot | Absolute monocyte count | TNF | LPS | 18 | 0.24 |
